# Heavy Metal Pollution of Lakes along the Mid-Lower Reaches of the Yangtze River in China: Intensity, Sources and Spatial Patterns

**DOI:** 10.3390/ijerph10030793

**Published:** 2013-02-26

**Authors:** Haiao Zeng, Jinglu Wu

**Affiliations:** State Key Laboratory of Lake Science and Environment, Nanjing Institute of Geography and Limnology, Chinese Academy of Sciences, 73 East Beijing Road, Nanjing 210008, China; E-Mail: zenghaiao@niglas.ac.cn

**Keywords:** the mid-lower reaches of Yangtze River, geo-accumulation index, heavy metals, lake sediments

## Abstract

Lakes in the middle and lower reaches of the Yangtze River form a shallow lake group unique in the World that is becoming increasingly polluted by heavy metals. Previous studies have largely focused on individual lakes, with limited exploration of the regional pattern of heavy metal pollution of the lake group in this area. This paper explores the sources, intensity and spatial patterns of heavy metal pollution of lake sediments. A total of 45 sample lakes were selected and the concentrations of key metal elements in the sediments of each lake were measured. The cluster analysis (CA), principal component analysis (PCA) and Geo-accumulation index (I_g_) analysis permitted analysis of the source and pollution intensity of the target lakes. Results suggested a notable spatial variation amongst the sample lakes. Lakes in the upper part of the lower reach of the Yangtze River surrounded by typical urban landscapes were strongly or extremely polluted, with high concentrations of Pb, Zn, Cu and Cd in their sediments. This was attributed to large amount of untreated industrial discharges and municipal sewage produced within the lake catchments. In contrast, the heavy-metal pollution of lakes in the Taihu Delta area was notably lower due to industrial restructuring and implementation of effective environmental protection measures. Lakes along the middle reach of Yangtze River surrounded by agricultural areas were unpolluted to moderately polluted by heavy metals overall. Our results suggested that lakes in the central part of China require immediate attention and efforts should be made to implement management plans to prevent further degradation of water quality in these lakes.

## 1. Introduction

Heavy metals such as cadmium, lead, copper and zinc are principal pollutants of aquatic ecosystems because of their environmental persistence, toxicity and great potential of accumulation in the food chains [1,2,3]. They enter the aquatic system through river flow or atmospheric deposition, and can be transported to the sediments immediately through absorption and sedimentation processes by suspended matters [4,5]. Thus, sediments are recognized as important sinks of heavy metals and they reflect the quality of an aquatic system. Because lakes are important sinks for many pollutants derived from their watershed, the lake sediments can provide rich information not only on the lake heavy metal pollution but also on environmental changes in the surrounding catchments [6,7,8,9].

The Yangtze River is the third largest river in the World and the longest river in China, with a mainstream length of over 6,300 km and a basin area of 1,800,000 km^2^, and it has 996 billion cubic meters of total water resources. The middle and lower reaches of the Yangtze River are located in the central area of China with freshwater shallow lakes and many lakes are important freshwater sources [10,11]. However, with the rapid population increase and economic growth, the water environment of the lakes has been deteriorating [12]. The lakes along the mid-lower reaches of Yangtze River constitute a shallow lake group that is unique in the World. It is a perfect region to explore the universal principles of environmental pollution in shallow lakes, which is useful to lake management [13]. Previous studies of lakes along the middle and lower reaches of the Yangtze River have largely focused on the excessive inputs of nutrients related to eutrophication [14,15,16]. Recent years have witnessed an increasing interest in the heavy metal pollution of some lakes, such as Cao Lake [17], Tai Lake [18], Poyang Lake [19], Dong Lake and Moshui Lake [20], *etc.* The studies have reported significant variations of heavy metal pollution among these lakes, but the regional pattern of the pollution remains unclear. Similar studies on lake groups with the same characteristics in other parts of the World involved few lakes. This study aims to investigate the spatial patterns of heavy-metal sources and pollution intensity in the middle and lower reaches of the Yangtze River area based on statistical analysis of 45 sample lakes. 

## 2. Material and Methods

### 2.1. Study Area

**Figure 1:**
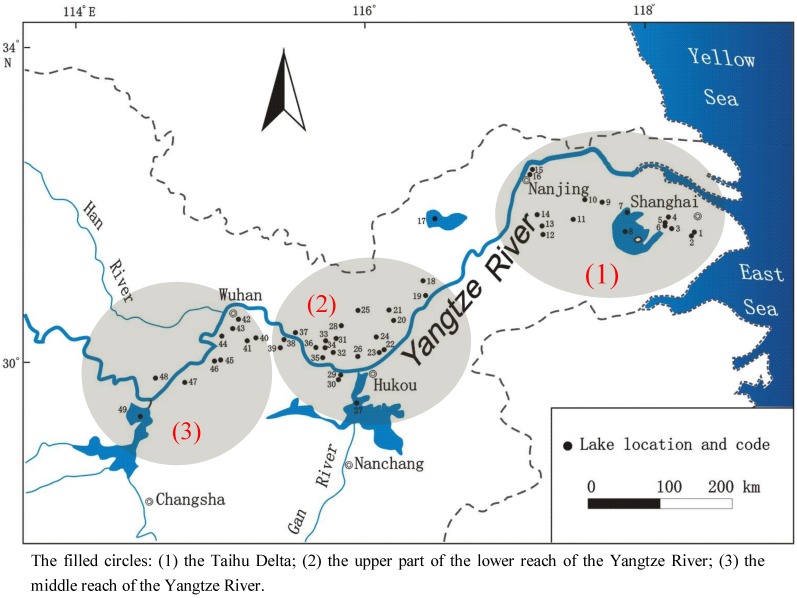
Location of sampled lakes along the middle and lower Yangtze River.

The Yangtze River was divided into three parts in terms of topographic and hydrologic characteristics. The upper stream of the river is from the source to Yichang in Hubei Province with a length of 4,510 km, where the famous 209 km long “Three Gorges” is located. From Yichang to Hukou in Jiangxi Province, the river enters the middle reach, with a length of 940 km. Down from Hukou is the lower reach with a length of 850 km. The middle and lower reaches of the Yangtze River are situated in the central and eastern parts of China, which feature the country’s most highly developed and densely populated areas. It also has the highest density of lakes, characterized by shallow and large surface area. Total lake surface area in this area is above 21,000 km^2^, accounting for 25% of the total lake surface area in China [11]. The lakes were all formed along with the evolution, flooding events and main-channel shifts of Yangtze River, with intense water and material exchange with the river [13]. Due to the rapidly expanding township enterprises, large population densities, the general over-use of agrochemicals and chemical fertilizers, the discharge of municipal sewage, and large-scale cultivation, most lakes in this area are under mesotrophic or eutrophic conditions [12]. This study selected 45 typical lakes to explore the spatial pattern of heavy metal pollution in the target area of the middle and lower reaches of the Yangtze River area (Figure 1).

### 2.2. Sampling and Chemical Analysis

Two or three sites were sampled in each lake depending on the size of the lake surface area. The samples were located in the sedimentary center to represent the average values of the respective lakes. Sampling of all lakes required two years (2003 and 2004) to complete because of the wide geographic distribution of the sampled lakes. Approximately the top 1 cm of sediments were collected using a piston-percussion corer fitted with 60 mm internal diameter perspex tubes. Immediately after recovery, each sample was sealed in a labeled plastic bag [21]. Sediment samples were oven-dried at 40 °C, ground in a mortar and pestle to <74 μm grain size, and then dried again at 105 °C prior to laboratory analyses. An accurately weighed sediment sample (~125 mg) was placed in a Teflon nitrification tank, 6.0 mL HNO_3_, 0.5 mL HCl, and 3.0 mL HF were added. The sealed tank was then placed in a microwave oven (Berghof MWS-3 Digester, Berghof, Germany) and nitrified at 180 ± 5 °C for 15 min. The residue from the tank was then transferred into a Teflon breaker and dissolved with 0.5 mL HClO_4_ in a heating block at about 200 °C and diluted to 25 mL with double distilled de-ionized water. The solution was then analyzed for elements by inductively coupled plasma-atomic emission spectrometry (ICP-AES). Standard EPA method 3052 was used for the determination of element concentrations [22]. The accuracy of the analytical determination was established using the reference material of GSD-9 and GSD-11, supplied by the Chinese Academy of Geological Sciences. The analytical results for all elements were found to be in agreement with the certified values, with measurement errors <5% [23]. 

### 2.3. Multivariate Analysis

Cluster analysis (CA) was performed to further classify the elements into groups representing different sources on the basis of similarities in their chemical properties [24]. Hierarchical cluster analysis for identifying relatively homogeneous groups of variables were selected in this study by using an algorithm that starts with each variable in a separate cluster and combines clusters until only one is left. Before CA, the variables were standardized by means of Z-scores; then Euclidean distances for similarities in the variables were calculated. A dendrogram was constructed to assess the cohesiveness of the clusters formed, in which correlations among elements can readily be seen.

Principal component analysis (PCA) is the most common multivariate statistical methods used to explore associations and origins of trace elements [25,26]. PCA is generally employed to reduce the dimensionality of a dataset while attempting to preserve the relationships present in the original data. Many researchers have used PCA in the evaluation of environmental data, obtaining interesting conclusions that are not immediately obvious. The concentrations of the trace elements evaluated in this study vary by different orders of magnitude. Thus, each variable was normalized to unit variance. After checking the suitability of the dataset for factor analysis, PCA with Varimax rotation was run. Only components with Eigenvalues greater than one unit after rotation were retained. In this study, PCA was conducted using SPSS, Version 18 for Windows XP.

## 3. Results

### 3.1. Metal Concentrations

Table 1 shows the statistical summary for the concentrations of elements in sediments in the study area. The element concentrations were the average concentrations of all samples in each lake. The mean concentrations of metal elements in the 45 lakes are in order of Al > Fe > K > Ca > Mg > Ti > Mn > Ba > Zn > V > Sr > Cr > Cu > Pb > Ni > Co > Be. Skewness and Kolmogorov-Smirnov test (K-S test) indicate that Al, Ba, Be, Co, Cr, Fe, K, Mg, Mn, Ni, Ti and V approach a normal distribution. On the contrary, the elements including Ca, Cu, Pb, Sr and Zn are positively skewed toward the lower concentrations, as also confirmed by the fact that the median concentrations of these elements are much lower than their mean concentrations. Based on the relative standard deviation (RSD) of the elements concentrations, the elements can be classified into three groups: Al, Ba, Be, Co, Cr, Fe, K, Mg, Mn, Ni, Ti and V with RSDs lower than 50%; Pb and Sr with RSDs between 50% and 100%; Cu and Zn with RSDs higher than 100%. The results of skewness, K-S test and RSD calculations suggest that the concentrations of some elements in the sediments at different lakes in the study area exhibit great spatial variability.

**Table 1:** Metal concentrations of lake sediments along the middle and lower reaches of Yangtze River.

Element	Unit	Minimum	Maximum	Median	Mean	S.D	RSD(%)	Skewness	K-S test	MDL
Al	mg/g	51.0	104.0	79.3	78.6	14.7	18.7	−0.092	0.786	0.02
Ba	mg/kg	291	805	533	523	93.8	17.9	0.449	0.867	1
Be	mg/kg	1.4	3.5	2.7	2.6	0.5	19.2	−0.539	0.573	0.1
Ca	mg/g	2.4	164.0	8.4	17.9	27.8	155.3	3.871	0.001	0.01
Co	mg/kg	5	52	22	22	8	36.4	1.274	0.734	1
Cr	mg/kg	50	159	101	103	29	28.2	0.331	0.624	1
Cu	mg/kg	21	1,462	43	88	218	247.7	5.986	0.000	1
Fe	mg/g	26.2	84.6	48.5	48.7	11.8	24.2	0.447	0.570	0.01
K	mg/g	10.3	25.6	19.4	18.8	3.4	18.1	−0.365	0.928	0.03
Mg	mg/g	4.0	18.6	8.4	8.8	3.3	37.5	1.209	0.481	0.01
Mn	mg/kg	508	2,365	1,243	1,303	450	34.5	0.463	0.667	0.5
Ni	mg/kg	20.4	81.0	45.0	46.1	13.1	28.4	0.399	0.876	1
Pb	mg/kg	24	166	43	50	27	54.0	2.512	0.010	2
Sr	mg/kg	41	658	94	115	93	80.9	4.903	0.001	0.5
Ti	mg/g	2.3	6.8	5.2	5.3	0.9	17.0	−0.752	0.799	1
V	mg/kg	62	185	127	126	31	24.6	−0.055	0.879	2
Zn	mg/kg	78	1,182	124	173	179	103.5	4.518	0.001	1

### 3.2. Multivariate Analysis Results

#### 3.2.1. Cluster Analysis

**Figure 2:**
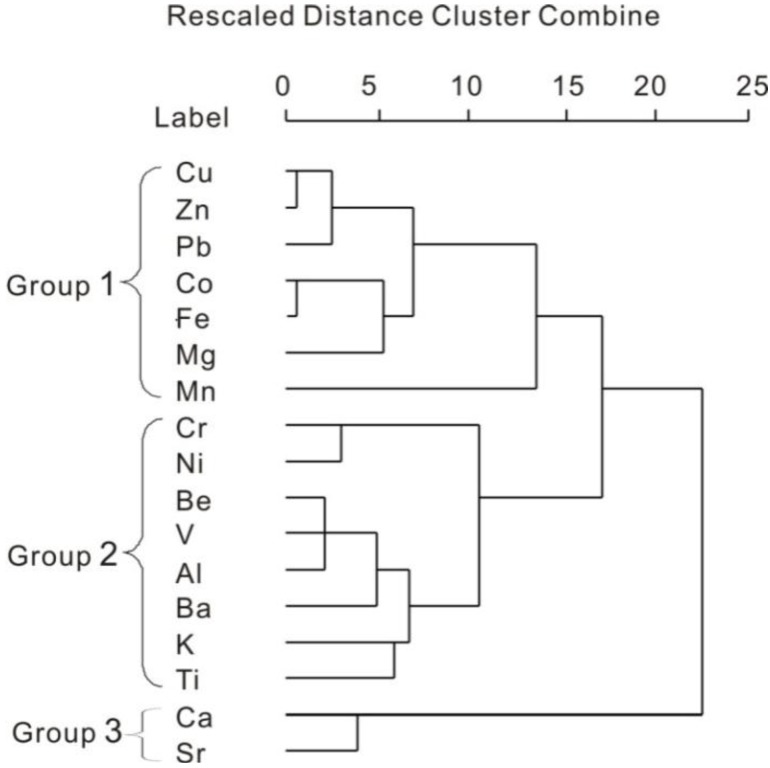
Dendrograms produced by hierarchical clustering of trace metals in the sediments from selected lakes.

CA was performed on the concentrations of metals in the lake sediments along the Yangtze River. The results are illustrated in the dendrogram (Figure 2). The distance cluster represents the degree of association between elements. The lower the value on the distance cluster, the more significant is the association. A criterion for the distance cluster of from 15 was used in the analysis. In the lake sediment, three distinct groups can be identified (Figure 2). Group 1: contained Cu, Zn, Pb, Co, Fe, Mg, Mn; These elements probably came from anthropogenic sources in urban areas. Group 2: contained Cr, Ni, Be, V, Al, Ba, K and Ti, These elements may originate from the natural parent materials of the soils. Group 3: contained Ca and Sr.

#### 3.2.2. Principal Components Analysis

**Figure 3:**
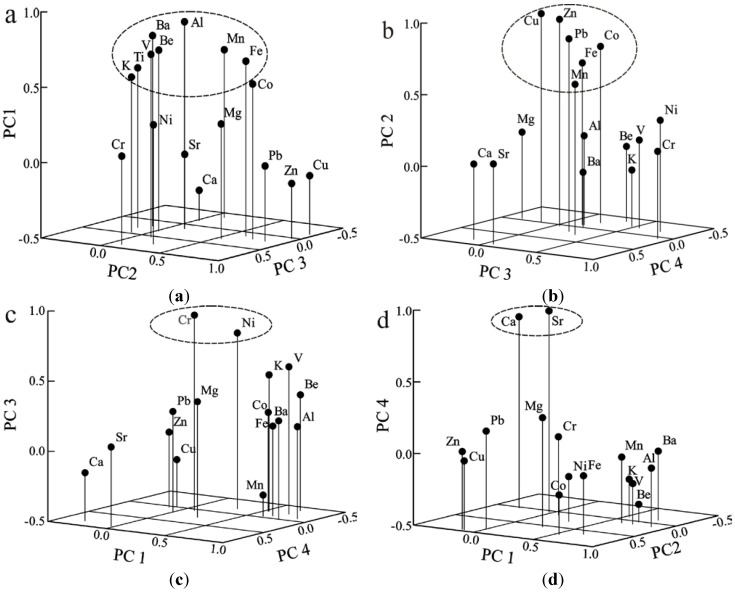
Loadings of the rotated eigenvectors, vertical axis are the relevant component: PC1, PC2, PC3 and PC4.

PCA was applied to assist in the identification of the sources of the trace elements. According to the Kaiser criterion, the first four components with eigenvalues are larger than 1.0 have dominant influences. The four principal components contribute 76.9% of the total variance in the samples (PCA output matrix not shown). The four principal components are shown by a 3D diagram in Figure 3. The first principal component (PC1) is characterized by Al, Ba, Be, Co, Fe, K, Mn, Ti and V (loading range 0.531–0.877), accounting for 27.34% of the total variance (Figure 3(a)). Principal component 2 (PC2), mainly dominated by Cu, Zn, Pb and Co (loading range 0.732–0.953), to a lesser extent by Fe (0.629) and Mn (0.549), accounts for 22.03% of the total variance (Figure 3(b)). 

As shown in Figure 3(c), Principal component 3 (PC3) is dominated by Cr (0.893) and Ni (0.752), accounting for 9.47% of the total variance. Principal component 4 (PC4) is characterized by high loadings for Ca (0.834) and Sr (0.907), accounting for 7.82% of the total variance (Figure 3(d)).

## 4. Discussion

### 4.1. Distribution of Heavy Metals

According to the PCA and CA analysis, the elements can be classified into four groups in terms of their sources and transport paths: group one (Cu, Zn, Pb, Co, Mn and Fe) were generally originated from anthropogenic activities and enter into the lakes through water flow. Group two (Cr and Ni) were generated from mixed sources. Group three (Be, V, Al, Ba, K, Ti, Mn and Fe) were mainly generated from natural processes. Group four (Ca and Sr) are not heavy metals. This study selected heavy metals in group one and two because they both were from anthropogenic activities and can indicate anthropogenic impacts on lake water pollution. Group three and four were excluded from the analysis. Therefore, Pb, Zn, Cu, Cr, Ni and Co were chosen to indicate the heavy metal pollution intensity of sediments in the sample lakes. 

**Figure 4:**
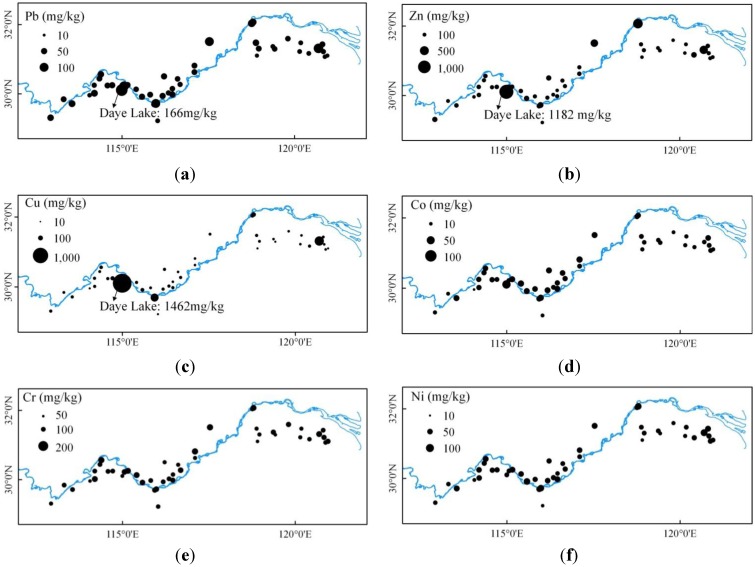
Spatial distribution of heavy metals of the lakes along the middle and lower reaches of Yangtze River.

The concentrations of the above heavy-metal indicators display notable spatial pattern. Figure 4 shows that the Pb, Zn, and Cu concentrations of lakes near urban areas are higher than the rest. For example, Lake (No. 39) is polluted by mining activity near the city of Huangshi and displays the highest concentrations of Pb (166 mg/kg), Zn (1,182 mg/kg), and Cu (1,462 mg/kg) among all the lake sediments. Jinji Lake (No. 5) is located in a special industrial zone in Suzhou City. By receiving waste water from the nearby factories, it presents high concentrations of Pb, Zn and Cu at 107 mg/kg, 431 mg/kg, and 370 mg/kg, respectively. Other lakes near urban areas, such as Xuanwu Lake, Mochou Lake, Bali Lake and Ci Lake, have high concentrations of Pb, Zn and Cu. Today much of the anthropogenic Cu, Zn and Pb originates from smelters, fossil fuel uses, industrial discharges, mining and wastewaters [27,28]. Heavy metals have been used by humans for a variety of purposes throughout the 20th century. The results suggested that the urban and industrial activities have made great contributions to heavy metal inputs. 

In addition to the above mentioned urban-rural differences, the concentrations of heavy metals also demonstrated remarkable regional patterns. Three distinguishable sub-regions of the middle and lower reaches of Yangtze River were identified, namely the middle reach of Yangtze River, the upper part of the lower Yangtze River, and the Taihu Delta. Lakes along the upper part of the lower Yangtze River generally have higher concentrations of heavy metals than the middle reach of Yangtze River and the Taihu Delta, with mean concentrations of Pb, Zn, Cu, Cr, Co and Ni reaching 58 mg/kg, 206 mg/kg, 134 mg/kg, 103 mg/kg, 19 mg/kg and 49 mg/kg, respectively. The equivalent values of Taihu Delta declined to 43 mg/kg, 163 mg/kg, 59 mg/kg, 93 mg/kg, 17 mg/kg and 44 mg/kg, respectively. The concentrations of the six elements in the middle reach of the Yangtze River were marginal in comparison with the upper part of the lower Yangtze River and Taihu Delta, except for some urban lakes, averaging at 47 mg/kg, 128 mg/kg, 47 mg/kg, 103 mg/kg, 19 mg/kg and 44 mg/kg, respectively. 

### 4.2. Source of Lake Sediments

From the descriptive statistical analysis, the concentrations of Ca, Sr, Cu, Pb and Zn exhibited great spatial variability in lake sediments, which suggests anthropogenic sources of these elements, while Al, Ba, Be, Co, Cr, Fe, K, Mg, Mn, Ni, Ti and V originate from natural sources. PCA and CA analyses are almost consistent with these interpretations, but the Ca and Sr group is remarkably different from the other elements, which implies a different origin from the other elements. Cr and Ni are correlated with Be, V, Al, Ba, K and Ti in CA, but separated from all of other elements in PCA. In addition, Fe and Mn were classified with heavy metal including Cu, Pb, Co and Zn, but had high score in both PC1 and PC2. The discrepancy indicated that the distributions of Cr, Ni, Fe and Mn were influenced by mixed factors. Previous studies also had found that Fe and Mn were more sensitive to redox-reactions than the other elements and showed high concentrations in the surface sediments in many lakes [27].

Based on PCA and CA, main sources with corresponding cluster elements can be identified. PC1 represents natural sources as it includes two conservative elements Al and Ti originated mainly from natural processes such as soil erosion [29]. PC2 can be defined as industrial sources as it is mainly constituted by Pb, Zn and Cu, which are commonly detected in industrial waste waters. PC3 might be associated with atmospheric deposition because the dominant element Cr enters the water body mainly through deposition [30]. PC4 can be classified as an authigenic source since it contains Ca and Sr which are dominant elements in grass-type lakes. 

**Figure 5:**
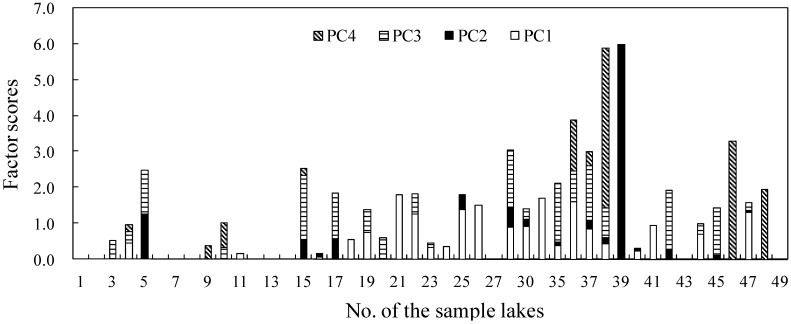
Spatial distribution of factor scores from PCA on the concentrations of elements (Negative scores not shown on the map). Lakes the Taihu Delta include No. 1 to No. 16; Lakes in the upper part of the lower Yangtze River include No. 17 to No. 39; Lakes in the the middle reach of the Yangtze River include No. 40 to No. 49.

The 45 sample lakes were scored for each principal component to identify their source of heavy-metal pollution. Figure 5 shows that lakes in the upper part of the lower Yangtze River have notably higher total scores in comparison with the middle reach of the Yangtze River and the Taihu Delta, further confirming the severe pollution in this area. 

Compared with the other two, lakes along the upper part of the lower Yangtze River generally have higher PC2 scores, indicating that the heavier pollution can be attributed to industrial discharges. For example, the highest PC2 score (5.99) occurs in Daye Lake (No. 39), possibly because it receives large amounts of waste water from the nearby mining sites. Cao Lake (No. 17) and Bali Lake (No. 30) also have a PC2 score above 0.5 due to the agglomeration of industrial zones around the two lakes. This result suggested that the upper part of the lower Yangtze River might need to tighten the existing control policies on industrial waste water treatment and release to prevent further degradation of the lake water environment. In addition, some lakes of the middle reach Yangtze River and the Taihu Delta surrounded by urban landscape also have high PC2 scores, for example, Jinji Lake (No. 5) and Xuanwu Lake (No. 15), resulting from untreated industrial waste water produced within the lake catchments, due to the large magnitude of industry wastewater loading. The results confirmed our conclusion that variations in heavy metal pollution exist not only amongst the three sub-regions, but also between urban and rural lakes.

### 4.3. Pollution Intensity of Heavy Metals

The geoaccumulation index (I_g_) was employed to evaluate the pollution intensity of each heavy metal in the sample lakes [6]:

I_gi _= log_2_ (C_i_/1.5B_i_)

where I_gi_ is the geoaccumulation index of heavy metal i; C_i_ is the measured concentration of heavy metal i in the lake sediment; and B_i_ is the geochemical background concentration of the metal. The factor 1.5 is used because of possible variations in background values due to lithological variability. The I_g_ is associated with a qualitative scale of pollution intensity. Samples may be classified as unpolluted (I_g_ ≤ 0), unpolluted to moderately polluted (0 ≤ I_g_ ≤ 1), moderately polluted (1 ≤ I_g_ ≤ 2), moderately to strongly polluted (2 ≤ I_g_ ≤ 3), strongly polluted (3 ≤ I_g_ ≤ 4), strongly to extremely polluted (4 ≤ I_g_ ≤ 5), and extremely polluted (I_g_ ≥ 5). In this study, based on the geographic location of each lake, the soil background values of Jiangsu, Anhui, Jiangxi, Hubei, and Hunan Province (Table 2), proposed by the China National Environmental Monitoring Center [31], were taken as the reference values to calculate the I_g_.

**Table 2:** Background values of heavy metals (mg·kg^−1^) in each province where these lakes are located.

Province	Cd	Cu	Pb	Zn	Co	Ni	Cr
Jiangsu	0.126	22.3	24.9	62.6	13.6	26.7	77.8
Anhui	0.097	20.4	26.9	62.0	16.3	29.8	66.5
Jiangxi	0.108	20.3	30.4	69.4	11.5	18.9	45.9
Hubei	0.172	30.7	27.1	83.6	15.4	37.3	86.0
Hunan	0.126	27.3	29.6	94.4	14.6	31.9	71.4

**Figure 6:**
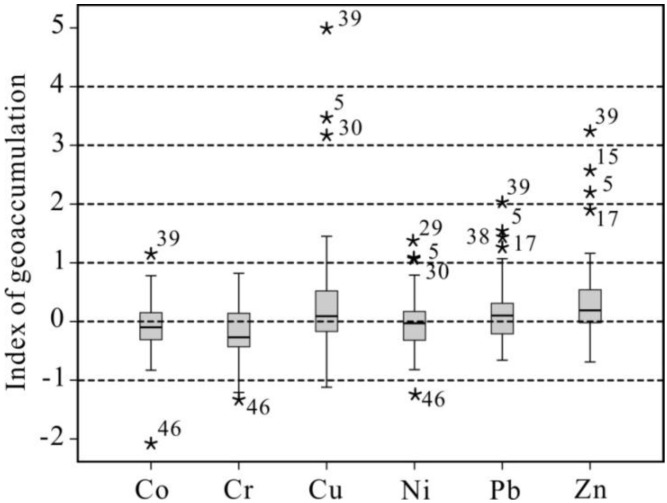
Boxplot of I_g_s of heavy metals in the lake sediments collected from the middle and lower reaches of Rangtze River. In this box plot, the horizontal line shows the mean values, the top and bottom of the box mark the limits of ±25% of the variable population, while the lines extending from the top and bottom of each box mark the minimum and maximum values that fall within 1.5 times the interquartile range below/above the 5th/95th percentiles. The stars represent I_g_s exceeding 1.5 times the width of the box.

Figure 6 shows that Cr, Ni and Co have mean I_g_s lower than zero, confirming its mainly natural source and are considered unpolluted. Pb, Zn and Cu with a mean I_g_s higher than 0, and with maximum I_g_s much higher than 3, were strongly polluted and considered to originate mainly from anthropogenic sources in this study. The mean I_g_s appears to display in the sequence of Zn > Cu > Pb > Ni > Co > Cr, which can also be seen as the decreasing order of their overall contamination degrees of lake sediments in the middle and lower reaches of Yangtze River. The I_g_ of Cd, Pb, Cu and Zn from Daye Lake sediment are 8.28, 2.03, 3.24, 4.99 respectively, which are extremely polluted by heavy metals. Other lakes around urban zones were moderately to strongly polluted by heavy metals, mainly from municipal drainage or industries that discharge waste. For example, I_g_ of Pb, Cu and Zn in Jinji Lake (No. 5) located in Suzhou city, are 1.55, 3.47, 2.20, respectively. Those of Xuanwu Lake (No. 15) located in Nanjing city, are 0.76, 1.45, 2.57, respectively.

**Figure 7:**
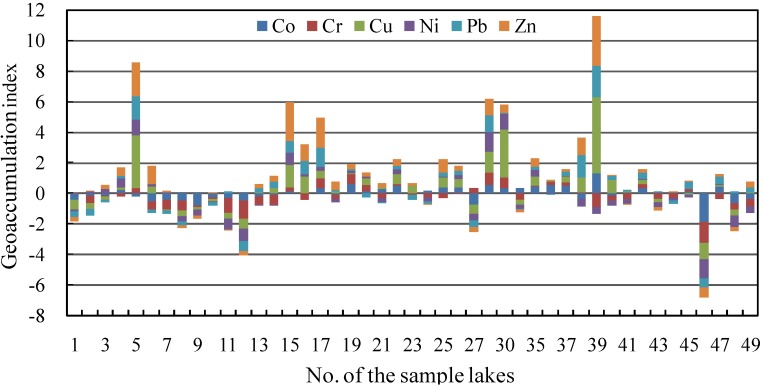
Spatial distribution of I_g_s of heavy metals in lakes along the middle and lower reaches of Yangtze River. Lakes in the Taihu Delta include No. 1 to No. 16; Lakes in the upper part of the lower Yangtze River include No. 17 to No. 39; Lakes in the middle reach of Yangtze River include No. 40 to No. 49.

Figure 7 shows that the pollution intensity of each heavy metal displays a similar spatial pattern with the heavy metal concentration and pollution source, with notable variations among the upper part of the lower Yangtze River, Taihu Delta and middle reach of the Yangtze River. 

Most of lakes along the upper part of the lower Yangtze River have notably higher I_g_s of Pb, Zn, Cu, maximizing at Daye Lake, with mean values of 0.59, 0.75 and 0.32, respectively, and could be considered as “moderately polluted” or “moderately to strongly polluted” by heavy metals. In contrast, despite rapid economic growth, the equivalent values of the Taihu Delta lakes declined to 0.45, 0.21 and 0.04, respectively. Most of the Taihu Delta lakes is considered to be “moderately polluted” by heavy metals, because of some environmental protection measures implemented there. Most of lakes along the middle reach of the Yangtze River have relatively low Igs values of Pb, Zn, Cu, with mean values of −0.03, −0.05 and 0.15, and are thus considered to be “unpolluted to moderately polluted” by heavy metals, respectively.

## 5. Conclusions

This study measured the concentrations of key heavy metal elements in 45 shallow lakes in the middle and lower reaches of the Yangtze River. Results show that most lakes in the area are facing pressing heavy metal pollution characterized by high accumulations of Cu, Zn and Pb in the lake sediments. The pollution intensity exhibits notable variations among the three distinguished sub-regions of our study area, namely the middle reach of the Yangtze River, the upper part of the lower Yangtze River, and the Taihu Delta. The pollution intensity is highest in the upper part of the lower Yangtze River, particularly in Anhui and Hubei Province due to intense anthropogenic activities. For instance, Daye Lake in the upper part of the lower Yangtze River is “extremely polluted” because of discharge of mining waste waters. Overall, the upper part of the lower Yangtze River belongs to the “moderately-polluted” group. Despite rapid economic growth, lakes in the Taihu Delta have relatively lower pollution than those in the upper part of the lower Yangtze River due to industrial structure upgrades and implementation of some effective environmental protection measures. In comparison with the upper part of the lower Yangtze River and the Taihu Delta, the heavy-metal pollution in the middle reach of Yangtze River is marginal, somewhere between the “unpolluted” to the “moderately polluted” level. 

It is noteworthy that the measuring data used in this study were obtained ten years ago in 2003. To analyze the trends of heavy-metal pollution of the three sub-regions, we compared our results with findings based on the most recent monitoring data in the same areas. It is found that in the Taihu Delta some key heavy metals had experienced drastic decreases from 2003 to 2011. For instance, the Pb concentrations of Mochou Lake (No. 15) and Xuanwu Lake (No. 16) have dropped from 69 mg/kg to 43.8 mg/kg, and 63 mg/kg to 23.4 mg/kg, respectively [32]. In contrast, the heavy metal pollution in the upper part of the lower Yangtze River might have experienced a slight increase during the same period. Specifically, Taibai Lake (No. 32) and Longgan Lake (No. 26) had their Pb concentrations increased from 43 mg/kg to 48.6 mg/kg, and 46 mg/kg to 54.1 mg/kg, respectively [33]. The results strongly suggested that lakes in the upper part of the lower Yangtze River, which lies in the centre part of China, should be given special attention for any upcoming protection and management plans to prevent further water quality degradation. 

Due to the time-consuming process of obtaining the spatial and statistic data on urbanization levels of the lake-surrounding areas, we were not able to provide quantitative information on the impacts of urbanization on heavy metal pollution intensity. At the current stage, we mainly attempted to explore the spatial pattern of heavy metal pollution and explain the possible reasons for the spatial variability of pollution intensity. The future work might aim to acquire more specific socio-economic data of the lake buffering areas to facilitate a quantitative analysis of the relationship between urbanization and heavy metal pollution.

## References

[1] Copat C., Bella F., Castaing M., Fallico R., Sciacca S., Ferrante M. (2012). Heavy metals concentrations in fish from Sicily (Mediterranean Sea) and evaluation of possible health risks to consumers. Bull. Environ. Contam. Toxol..

[2] Farombi E.O., Adelowo O.A., Ajimoko Y.R. (2007). Biomarkers of oxidative stress and heavy metal levels as indicators of environmental pollution in African Cat Fish (*Clarias gariepinus*) from Nigeria Ogun River. Int. J. Environ. Res. Public Health..

[3] Opfer S.E., Farver J.R., Jeffrey G., Krieger M.K. (2011). Heavy metals in sediments and uptake by burrowing mayflies in western Lake Erie basin. J. Great Lakes Res..

[4] Deepulal P.M., Gireesh kumar T.R., Sujatha C.H., George R. (2012). Chemometric study on the trace metal accumulation in the sediments of the Cochin Estuary-Southwest coast of India. Environ. Monit. Assess..

[5] Dassenakis M., Andrianos H., Depiazi G. (2003). The use of various methods for the study of metal pollution in marine sediments, the case of Euvoikos Gulf, Greece. Appl. Geochem..

[6] Stéphane A., Jörg S., Gérard B., Jouanneau J.M. (2004). Fifty-year sedimentary record of heavy metal pollution (Cd, Zn, Cu, Pb) the Lot River reservoirs (France). Environ. Pollut..

[7] Thevenon F., Neil D., Chiaradia G.M., Arpagaus P., Wildi W., Poté J. (2011). Local to regional scale industrial heavy metal pollution recorded in sediments of large freshwater lakes in central Europe (lakes Geneva and Lucerne) over the last centuries. Sci. Total. Environ..

[8] Thevenon F., Guédron S., Chiaradia M., Loizeau J.L., Poté J. (2011). (Pre-) historic changes in natural and anthropogenic heavy metals deposition inferred from two contrasting Swiss Alpine lakes. Quaternary Sci. Rev..

[9] Callender E., Rice K.C. (2000). The urban environmental gradient: Anthropogenic influences on the spatial and temporal distributions of lead and zinc in sediments. Environ. Sci. Technol..

[10] Wang S.R., Jin X.C., Niu D.L., Wu F.C. (2009). Potentially mineralizable nitrogen in sediments of the shallow lakes in the middle and lower reaches of the Yangtze River area in China. Appl. Geochem..

[11] Wang S.M., Dou H.S. (1998). Memoirs of Lakes in China.

[12] Qin B.Q. (2002). Approaches to mechanisms and control of eutrophication of shallow lakes in the middle and lower reaches of the Yangtze River. J. Lake Sci..

[13] Qin B.Q., Zhu G.W. (2006). The nutrient forms, cycling and exchange flux in the sediment and overlying water system in lakes from the middle and lower reaches of Yangtze River. Sci. ChinaEarth Sci..

[14] Wang S.R., Jin X.C., Zhao H.C. (2006). Phosphorus fractions and its release in the sediments from the shallow lakes in the middle and lower reaches of Yangtze River area in China. Colloid Surface Physicochem. Eng. Aspect..

[15] Wu J.L., Huang C.M., Zeng H.A., Schleser G.H., Battarbee R. (2007). Sedimentary evidence for recent eutrophication in the northern basin of Lake Taihu, China: Human impacts on a large shallow lake. J. Paleolimnol..

[16] Yao S.C., Li S.J. (2004). Sedimentary records of eutrophication for the last 100 years in Caohu Lake. Acta Sedimentologica Sinica..

[17] Tang W.Z., Shan B.Q., Zhang H., Mao Z.P. (2010). Heavy metal sources and associated risk in response to agricultural intensification in the estuarine sediments of Chaohu Lake Valley, East China. J. Hazard. Mater..

[18] Jiang X., Wang W.W., Wang S.H., Zhang B., Hu J.C. (2012). Initial identification of heavy metals contamination in Taihu Lake, a eutrophic lake in China. J. Environ. Sci..

[19] Yuan G.L., Liu C., Chen L., Yang Z.F. (2011). Inputting history of heavy metals into the inland lake recorded in sedimentprofiles: Poyang Lake in China. J. Hazard. Mater..

[20] Yang Z.F., Wang Y., Shen Z.Y. (2009). Distribution and speciation of heavy metals in sediments from the mainstream, tributaries, and lakes of the Yangtze River catchment of Wuhan, China. J. Hazard. Mater..

[21] Wu J.L., Li S.J., Luecke A., Wang S.M. (2002). Climatic signals in the last 200 years from stable isotope record in the shells of freshwater snails in Lake Xingcuo, eastern Tibet Plateau, China. Chin. J. Geochem..

[22] (1996). USEPA Method 3052: Microwave Assisted Acid Digestion of Siliceous and Organically Based Matrices.

[23] Zeng H.A., Wu J.L. (2009). Sediment records of heavy metal pollution in Fuxian Lake, Yunnan, China: The intensity, history and source. Pedosphere.

[24] Ragosta M., Caggiano R., Macchiato M. (2008). Trace elements in daily collected aerosol: Level characterization and source identification in a four-year study. Atmos. Res..

[25] Külahcl F., Şen Z. (2008). Multivariate statistical analyses of artificial radionuclides and heavy metals contaminations in deep mud of Keban Dam Lake, Turkey. Appl. Radiat. Isotopes..

[26] Loska K., Wiechuł D. (2003). Application of principal component analysis for the estimation of source of heavy metal contamination in surface sediments from the Rybnik Reservoir. Chemosphere.

[27] Maldonado V.M., Rubio Arias H.O., Quintana R., Saucedo R.A., Gutierrez M., Ortega J.A., Nevarez G.V. (2008). Heavy metal content in soils under different wastewater irrigation patterns in Chihuahua, Mexico. Int. J. Environ. Res. Public Health..

[28] Bertin C., Bourg A.C.M. (1995). Trends in the heavy metal content (Cd, Pb, Zn) of river sediments in the drainage basin of smelting activities. Water Res..

[29] Din Z. (1992). Use of aluminum to normalize heavy metals data from the estuarine and coastal sediments of the strait of Melaka. Mar. Pollut. Bull..

[30] Huang S.S., Tu J., Liu H.Y., Hua M., Liao Q.L., Feng J.S., Weng Z.H., Huang G.M. (2009). Multivariate analysis of trace element concentrations in atmospheric deposition in the Yangtze River Delta, East China. Atmos. Environ..

[31] China National Environmental Monitoring Center (1990). The Background Values of Chinese Soils.

[32] Bing H.J., Wu Y.H., Liu E.F., Yang X.D. (2010). The accumulation and potential ecological risk evaluation of heavy metals in the sediment of different lakes within the middle and lower reaches of Yangtze River. J. Lake Sci..

[33] Xin H., Wang C., Zou L.M. (2011). Characteristics of heavy metals and Pb isotopic signatures in sediment cores collected from typical urban shallow lakes in Nanjing, China. J. Environ. Manag..

